# Using an unmanned aerial system to analyse environmental impacts of charcoal production on tropical savanna ecosystems in northwestern Kenya

**DOI:** 10.1007/s10661-022-10241-2

**Published:** 2022-07-29

**Authors:** Maike Petersen, Marcus Nüsser

**Affiliations:** 1 Department of Geography, South Asia Institute, Heidelberg University, Heidelberg, Germany; 2 Heidelberg Center for the Environment, HCE, Heidelberg University, Heidelberg, Germany

**Keywords:** Charcoal production, Vegetation density, Unmanned aerial system, Remote sensing, Concentric ring analysis, Kenya

## Abstract

In many regions of Sub-Saharan Africa, charcoal plays an important role as energy source but is widely perceived as a major driver of deforestation and forest degradation. This narrative, however, is mostly based on research within primary production regions. Though space-borne remote sensing applications can be useful in monitoring such large-scale production modes, environmental effects of household-level production are less easy to assess. Therefore, the present study employs an unmanned aerial system (UAS) to assess the impact of small-scale charcoal production on the vegetation density in the immediate vicinity of production sites. The UAS data was complemented by field measurements and very high-resolution WordView-2 satellite imagery. This approach revealed only small differences between charcoal production sites and reference plots which were usually evened out after 20–25-m distance to the plot centre using a concentric ring analysis. Results further show that a distinction between different land-use practices is difficult, even with the high spatial resolution provided by a UAS. Thus, more research and new approaches are needed to evaluate the role of small-scale charcoal production in deforestation and forest degradation processes against the background of other human activities. However, to exploit the full potential of UAS for monitoring environmental effects in charcoal producing areas, official regulations need to be clearer and more reliable.

**Supplementary Information:**

The online version contains supplementary material available at 10.1007/s10661-022-10241-2.

## Introduction

Forest and savanna ecosystems in sub-Saharan Africa are characterized by significant changes in land cover and vegetation composition due to agro-pastoral activities and impacts from various other land-use practices (Arakwiye et al., 2021; Dendoncker et al., 2020; Ellis, 2006). In many regions of Sub-Saharan Africa (SSA), charcoal production plays an important role as a driver in environmental change. As it is valued by end users for its affordability, compactness, and high-energy storage, charcoal is the most important cooking fuel for urban households throughout SSA (Zulu & Richardson, 2013). The narrative of an energy ladder, where households use charcoal as a transitional state between traditional fuels such as firewood or dung, and modern energy sources such as electricity and liquefied petroleum gas (LPG) is increasingly questioned (Castán Broto et al., 2020; Yadav et al., 2021). Even though many urban households already use LPG, the majority relies on an energy mix including charcoal to even out seasonal variabilities in supply and price (Hiemstra-van der Horst & Hovorka, 2008). Recent studies agree that charcoal demand will continue to rise with ongoing urbanization (D’Agostino et al., 2015; Santos et al., 2017). For the case of Kenya, the Food and Agriculture Organization (FAO) predicts a continued population growth and urbanization for the coming decade together with a potential increase in charcoal demand and production volume (FAO, 2019; KFS, 2013). Nevertheless, several projects aim at reducing charcoal consumption by developing more efficient cookstoves (Ingwe-Musungu et al., 2014). Though these efforts are important and positively affect human health by reducing indoor air pollution, their impact on charcoal production is expected to be negligible in the face of future consumption increase (Arnold et al., 2006; Mehetre et al., 2017; Nakora et al., 2020; Njenga et al., 2016).

Predictions about a rising demand come with worries about adverse environmental effects. While greenhouse gas emissions (Ekeh et al., 2014; Pennise et al., 2001) and ambiguous impacts on soil properties (Abebe & Endalkachew, 2011) play a role in the debate on charcoal production, the focus is mostly on the severe degradation of wood resources (Mensah et al., 2020). Since the 1970s, researchers have been debating on the existence of a “wood fuel crisis” caused by the high demand for charcoal^1^ (Dewees, 1989; Eckholm, 1975). More recently, however, these views are increasingly questioned as studies in several areas found the environmental impact of charcoal production to be less distinct than previously thought (Chidumayo & Gumbo, 2013; Doggart et al., 2020; Petersen et al., 2021). In the context of deforestation, charcoal is rather regarded as a side product of agricultural expansion than the primary cause for the complete removal of trees (Iiyama et al., 2017; Mwampamba et al., 2013). Forest degradation through selective logging of preferred tree species for production appears to play a more important role (Hosonuma et al., 2012; Ndegwa et al., 2016). However, since most studies on charcoal production are located in primary production regions, the situation in remote, small-scale production areas is rarely considered in the debate around this economic sector. Consequently, the impacts of small-scale charcoal production on the environment are only poorly understood.

While monitoring deforestation processes is relatively straight forward, detection and quantification of forest degradation are more complex (Dupuis et al., 2020; Herold et al., 2011; Lambin, 1999; Shiferaw & Suryabhagavan, 2019). The spatial resolution of space-borne sensors such as Landsat or Sentinel (10–30 m) does not allow detailed analyses of small-scale processes of vegetation changes. Though Sedano et al. (2020) have been able to trace forest degradation in the context of charcoal production within a large-scale production area based on Landsat imagery, this approach is not suitable for smaller production areas where kilns are generally smaller. In addition to space-borne solutions, unmanned aerial systems (UASs) have emerged as an indispensable tool for environmental research over the past decade (Green, 2020; Whitehead & Hugenholtz, 2014). For scientific use, small fixed- or rotary-wing platforms are often equipped with RBG, multispectral or thermal cameras, or in some cases with other sensors such as lasers or anemometers (Berni et al., 2009; Nebiker et al., 2014; Prudden et al., 2018; Sankey et al., 2017; Valavanis & Vachtsevanos, 2015).

Such air-borne observation devices have been successfully employed to assess vegetation parameters (Goodbody et al., 2017; Kolarik et al., 2020), monitor damage from forest fires (Carvajal-Ramírez et al., 2019; de Roos et al., 2018), and observe vegetation recovery after disturbances (Larrinaga & Brotons, 2019; Talucci et al., 2020). However, research on the potential of these platforms to map environmental impacts of charcoal production remains limited. There are only few studies on tropical forests where timber is also extracted for charcoal production (Gobbi et al., 2020; Otero et al., 2018) but none specifically investigates vegetation changes in relation to production sites. Against this background, the present study is designed to test the potential of a UAS in monitoring small-scale charcoal production and its impact on vegetation structures in northwestern Kenya. The UAS has been used to evaluate effects on the vegetation density in one focus area with high production activity. The data has been complemented by field measurements and very high-resolution (VHR) WordView-2 satellite imagery. Thus, the following two questions are addressed:

- To what extent does the vegetation density in the vicinity of production sites differ from non-production reference sites?
- How can a UAS be utilized in monitoring environmental effects of charcoal production?

## Study area

**Fig. 1:**
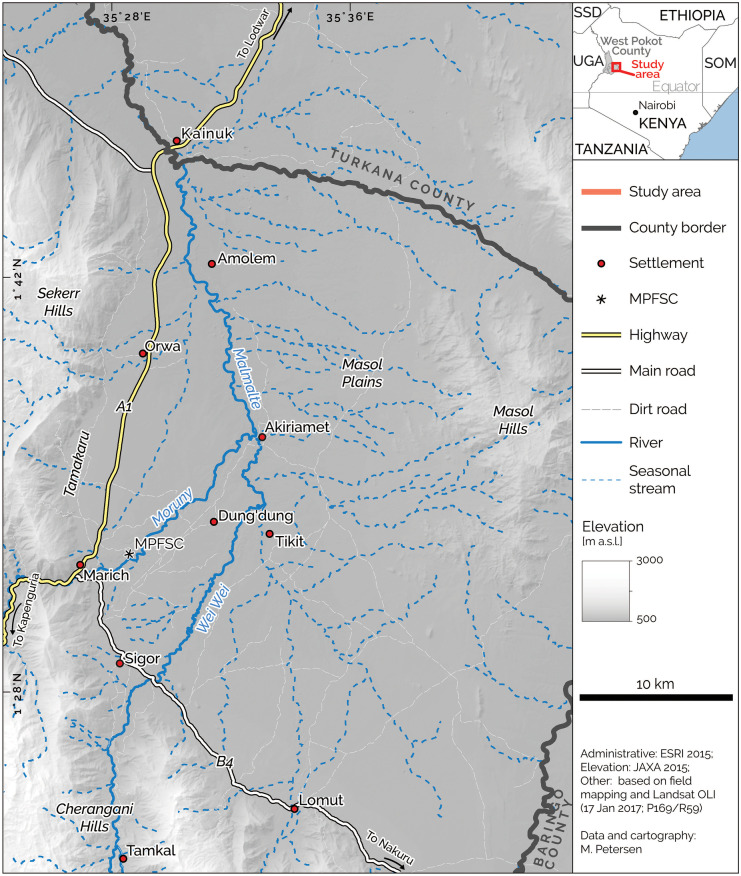
Study area in Pokot Central with the location of roads, settlements, and water sources. The study’s base was at Marich Pass Field Studies Centre (MPFSC)

The case study is located in the drylands of Pokot Central, an administrative sub-unit of West Pokot County (WPC) in northwestern Kenya (Fig. 1). The study area is characterised by semi-arid conditions with an annual precipitation of approximately 400 mm at high temporal and spatial variability (GeoInformatiks Ltd, 2017). Most of the area is covered by bush savanna predominantly consisting of different *Vachellia* species. The perennial rivers are lined by forests with tall, evergreen trees. To a smaller extent, these galley forests can be found in thin bands along seasonal streams and runoff channels. Patches of *thickets* dominated by *Euphorbia* spp. border the riverine gallery forests and have high vegetation densities of over 70%. Depending on density, the bush savanna can also be grouped into *sparsely vegetated* (below 15% vegetation cover), *open woodland* (15–40%), and *closed woodland* (40–70%). Settlements are clustered around a few centres such as Sigor, Orwa, and Marich, while many homesteads are scattered across the study area leading to a low population density (58 persons per km^2^, KNBS, 2019). Most houses are round and built from wooden poles, mud, and grass and only few houses are rectangular with corrugated iron roofs. Like in other rural areas of Kenya (Nkedianye et al., 2020; Roden et al., 2016; Verkaart et al., 2018), livelihoods in Pokot Central mainly depend on small-scale agriculture and pastoralism (WPC Government, 2018). Since the 1970s, the remote area is connected to urban centres in the south via the A1 Highway between Kapenguria and Lodwar (Fig. 1).

### Charcoal production in Pokot Central

Improved access to urban markets, displacement and resettlement of people affected by violent events in the 1990s are relevant factors in the rise of charcoal production in the study area. Socio-economic changes and environmental dynamics are interwoven, as intensive bush encroachment within the area has altered the availability of natural resources over the past 30 to 40 years (Conant, 1982; Petersen et al., 2021). Small-scale production is organized at household level with an average monthly output of approximately 500 kg per household (own assessments, field measurements) as compared to large-scale production sites where one producer yields over 3800 kg per month (Kambewa et al., 2007). As charcoal production is illegal in Pokot Central, no official records of sales and exports exist. Production hotspots are mostly located in the vicinity of the A1 Highway where charcoal is sold to drivers of empty lorries returning from South Sudan and Turkana towards Kapenguria, Kitale, and other urban centres further south where charcoal consumers are clustered (Bergmann et al., 2019).

## Methods

### Field and UAS survey

One characteristic focus area (1 × 1 km^2^) with a high density of charcoal production sites located between the settlements of Marich and Orwa was selected for the UAS surveys.^2^ This focus area was completely surveyed by foot in a wall-to-wall sampling. Current and former kilns or ash residues of former kilns (kiln burn marks, KBMs) were mapped and measured. In the immediate surrounding of these plots (approximately 50 m), additional information on land-use and land-cover class, tree species, and stumps were collected. Wherever possible, charcoal producers of respective kilns were interviewed about the time that had passed since the kiln had been in use and the amount of charcoal harvested (in number of bags^3^). Otherwise, the local research assistant estimated the age by assessing the KBM’s colour (Fig. SI 1d) and compactness, type, and age of plants covering the KBM (Fig. SI 1e), as well as the amount of larger charcoal pieces still present. The harvested amount of charcoal was deduced based on the KBM’s size as they were found to be strongly correlated (Dons et al., 2015). UAS flights were conducted from November 2018 to February 2019 using a quadcopter DJI Mavic Pro (DJI, 2021). The dry season was chosen for the UAS flights, to avoid interference from the herbal layer which quickly covers the ground after rainfall. By data acquisition in the dry season, vegetation density can largely be related to woody vegetation. The seasonal difference between kiln mapping and UAV flights did not affect the study, as only location and size of the kilns and KBMs were used in the analysis. Additional information on surrounding tree types, age of KBM, estimated charcoal output, etc. was mainly used to complement the study and to generate a more holistic understanding of regional charcoal production.

A total of 4215 images, covering an area of 1.16 km^2^, were collected via parallel flights on five days during January 2019, resulting in a ground resolution of 1.63 cm per pixel. The images were processed with the software Agisoft Metashape Professional 1.5 (Agisoft, 2019a) to reconstruct 3D-information from 2D-images (Kabiri et al., 2020; Kienholz et al., 2020; Thiel et al., 2020). This resulted in a digital surface model (DSM), a digital terrain model (DTM), and consequently in a normalized digital surface model (nDSM), showing the height of any object on the ground (for more detailed information see SI 1 and Fig. SI 2). Based on the DSM, the single images were mosaiced into one orthophoto of the focus area. Based on the orthophoto and ground data, footpaths and homesteads were mapped to better describe and understand the intensity of utilization in the focus area.

### Modelling vegetation density

Agisoft Metashape applied the structure from motion technique, which combines photogrammetric principles and computer vision. In a first step, images were aligned by reconstructing each image’s position and orientation based on EXIF information and image matching. In this process, corresponding feature points in overlapping images were identified and their disparity measured. Subsequently, photogrammetric principles were employed to extract three-dimensional information (Remondino et al., 2014; Agisoft, 2019b). Accuracy of this step was set to “medium,” to reduce processing time. Default key and tie point limits were adopted as 40,000 and 4000, respectively. The resulting sparse 3D cloud of tie points was improved manually, to increase accuracy. Objects were visually identified in different images and matched together. Thus, images that had not been aligned automatically were then included in further processing. Using this combined method, it was possible to align 4205 out of the total of 4215 images. Based on this initial output (2.5 Mio. points), a dense point cloud was built by a pixel-based multi-view stereo reconstruction (Verhoeven, 2011). The quality parameter for this step was set to “medium,” to limit processing time and mild filtering was applied to avoid elimination of relevant points. As no point data for validation was available, the point cloud was evaluated only visually. Resulting points were automatically classified into ground points and above ground points adopting default settings. In this step, the software divided the cloud into cells and searched for the lowest point within each cell. The initial terrain model was then complemented by additional points that lay within a certain distance and angle to the previously classified points (Becker et al., 2018; Agisoft, 2019b).

**Fig. 2:**
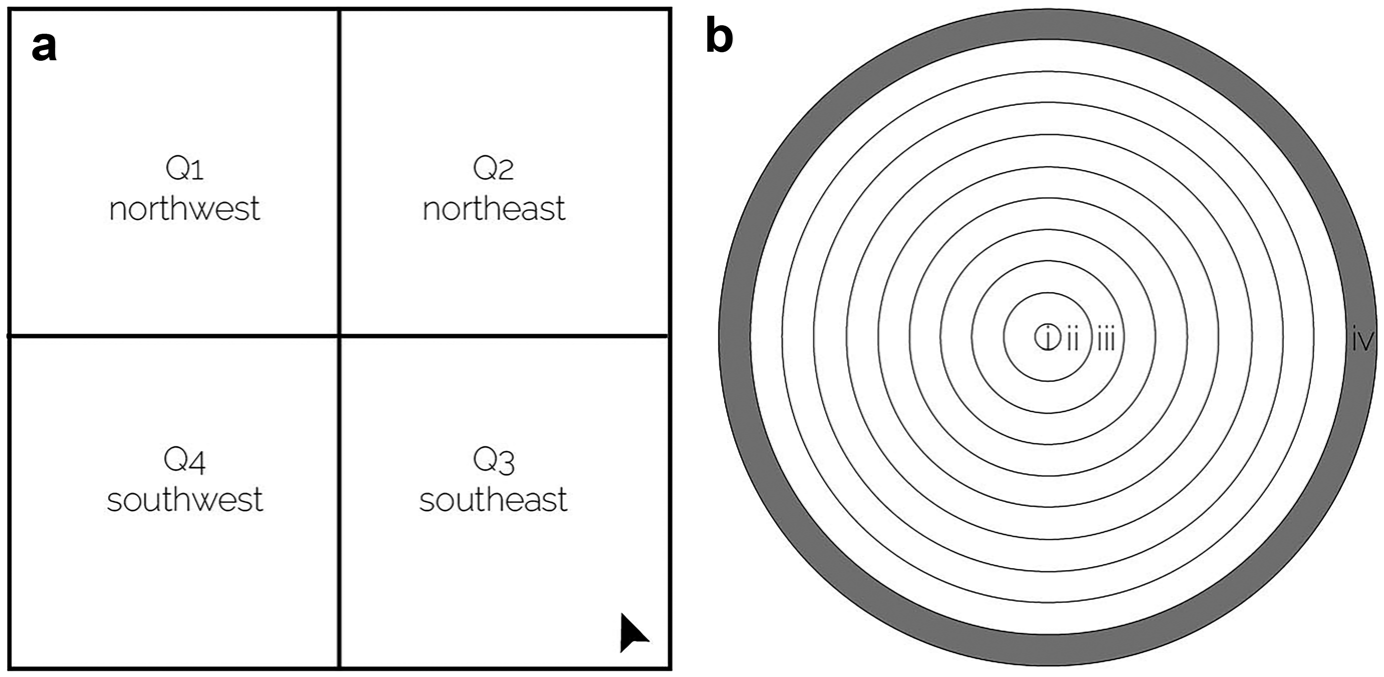
Schematics of assessing the impact on vegetation structures. **a** The focus area was divided into four quadrants to support comparison within it. **b** Ten concentric rings were drawn around each (KBM/reference or homestead) plot centre (i) and the mean vegetation density or the SAVI within each buffering (e.g. ii, iii, and iv) extracted from the base layer

The resulting dense point cloud of the focus area was further processed in ArcMap 10.6. The tool “las point statistics as raster” was used to produce two datasets. One for the number of points per cell which had been classified as ground points, thus representing non-vegetated areas. The second dataset showed the number of points per cell classified as non-ground points, representing features which are located higher than the ground. The cell size was set to four times the average point spacing (the minimum value). Dividing the above-ground point count by the total point count generated a dataset with values ranging from 0 to one representing the vegetation density of each 4-m raster cell. This was further classified into density classes in accordance with land-use and land-cover (LULC) classifications from Petersen et al. (2021). However, the higher spatial resolution allowed subdivision of woodland into open woodland and closed woodland resulting in four LULC classes used in the present study (sparsely vegetated, open woodland, closed woodland, and thicket). All outputs were clipped to the 1 km^2^ extent of the focus area. This was furthermore subdivided into four quadrants (Q1, Q2, Q3, Q4) to allow description and comparison of the study site (Fig. 2a).

### Concentric ring analysis of vegetation density

To assess the impact of charcoal production on vegetation structure, the vegetation density model of the 1 km^2^ focus area was combined with field measurements and GPS points of all kilns and KBMs. A buffer was added to each kiln/KBM point; its size represented its size as measured in the field (Fig. 2b-i). The resulting polygon was assigned the LULC class it was located in. Subsequently, ten concentric rings were added in 5-m steps. Thus, the first ring (Fig. 2b-ii) around the centre describes the first 5 m around the kiln/KBM, and the second ring (Fig. 2b-iii) represents the area between 5 and 10 m around the centre. The last buffer ring (Fig. 2b-iv) demarcates a distance between 45 and 50 m to the kiln/KBM.

A spatial join then assigned each of the concentric rings the mean value of the underlying vegetation density cells. The same number of reference plots, where no kiln or KBM had been detected, was processed accordingly. Furthermore, all homesteads in the study area were analysed similarly. The centre of each homestead was identified as the geographical centre of all features (hut, livestock enclosure, and structures for storage or kitchen). The size of the first buffer was set to 18 m which was the mean size of all homesteads. This value, however, is based on estimations, as an exact delineation of homesteads is not practicable. Results were then transferred to MS Excel and analysed descriptively based on the LULC class at each plot centre.

### Concentric ring analysis of the soil adjusted vegetation index

To compare the results from the UAS survey, the concentric ring analysis was repeated. Instead of the UAS-derived vegetation density, it was based on soil-adjusted vegetation index (SAVI) values with a soil coefficient *L* = 0.5. The SAVI accounts for the spectral variability of background materials which makes it a robust index for dryland ecosystems with scattered vegetation cover (Barati et al., 2011; Huete, 1988). The SAVI was calculated from a tasked WorldView-2 scene (30 Jan 2019, 0.5-m resolution). The resulting layer was analysed following the workflow described above, producing a similar output which related to the photosynthetic activity of the vegetation instead of the density. Including this dataset in the analysis allowed to validate the UAS data and compared air- and space-borne approaches.

## Results

**Fig. 3:**
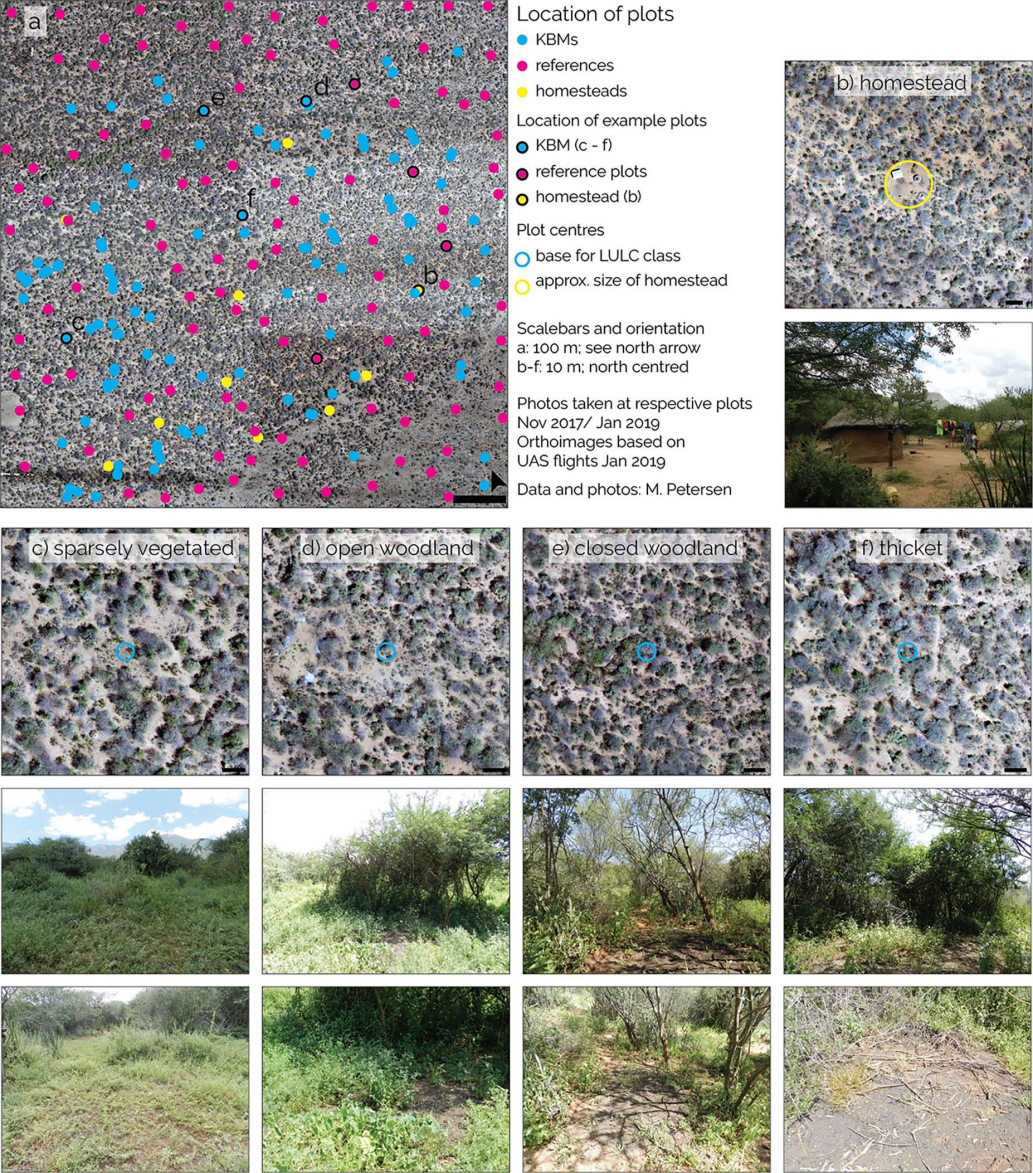
Distribution of KBMs, homesteads, and reference plots within the focus area. **a** distribution of the plot centres across the focus area, the examples **b**–**f** are indicated as circled dots. **b** Aerial view and ground photo from a homestead. **c**–**f** Aerial view and ground photo of KBM and surroundings at KBM plots in the four LULC classes, sparsely vegetated (**c**), open woodland (**d**), closed woodland (**e**), and thicket (**f**). Note that ground images were taken shortly after rainy season and UAS image acquisition was during dry season, which results in more herbal vegetation cover in the ground photos

Image matching resulted in a DSM (Fig. SI 3b) with elevations between 925 and 970 m a.s.l., a pixel size of 10 cm and a point density of 100 points per m^2^. Classification of the point cloud into ground points allowed generating a DTM (between 920 and 970 m a.s.l.; Fig. SI 4c) and a subsequent subtraction of the DTM from the DSM produced an nDSM (Fig. SI 3d) with heights between 0 and 14 m a.g.l. (above ground level). The overall root mean square error of the ground control points was calculated at 2.5 m with a latitude/longitude error of 1.9 m and 1.6 m in *z*-direction. Vegetation density based on the proportion of above-ground points to total points per pixel resulted in a mean vegetation density of 24% (Fig. SI 3e). The dominant LULC class based on vegetation density is sparsely vegetated (43%), followed by open woodland (26%), closed woodland (21%), and thicket (10%), which can be mostly found in the centre of the scene and along seasonal river channels which were detected in the DTM (Fig. SI 3f). Ten homesteads consisting of one to three buildings and usually a livestock enclosure on a small piece of cleared land are located within the focus area as well as three abandoned huts. Depending on family size and resources, a separate building was used as a kitchen and other structures such as storage constructions or sheds for livestock exist. Eight of the currently used homesteads are located in the southern half (Fig. 3a and b). The focus area is crossed by 12.6 km of detectable footpaths, frequently used by its residents, motorbike taxis, and livestock.

### Distribution of kilns and kiln burn marks

During the field mapping on November 2017, a total of 105 kilns/KBMs were detected, mapped, and measured within the focus area. Five additional kilns/KBMs were added during the UAS survey on January 2019. The mean size of the KBMs ash residues is 11.1 m^2^ (median: 9.3 m^2^) with a mean estimated age of 22.5 months (median: 18 months) in relation to January 2019. The mean output was approximated at 3.8 bags per kiln (median: 3). Only one active kiln was detected and its KBM size was projected based on the conversion rate, *CR*_*k*_ = 3.5, which was derived from field measurements.

Kilns/KBMs are unevenly distributed across the study area with most of them (47 of 110) clustered in the southwestern and fewest (15) in the northwestern quadrant (Fig. 3a). Most kilns/KBMs can be found within the LULC class of sparsely vegetated (61%), within open woodland (22%) as well as in closed woodlands (13%), while thickets hold the fewest number (5%). Figure 3c–f gives an example for a KBM located within each LULC class.

### Vegetation characteristics in relation to the distance from kiln burn marks

**Fig. 4:**
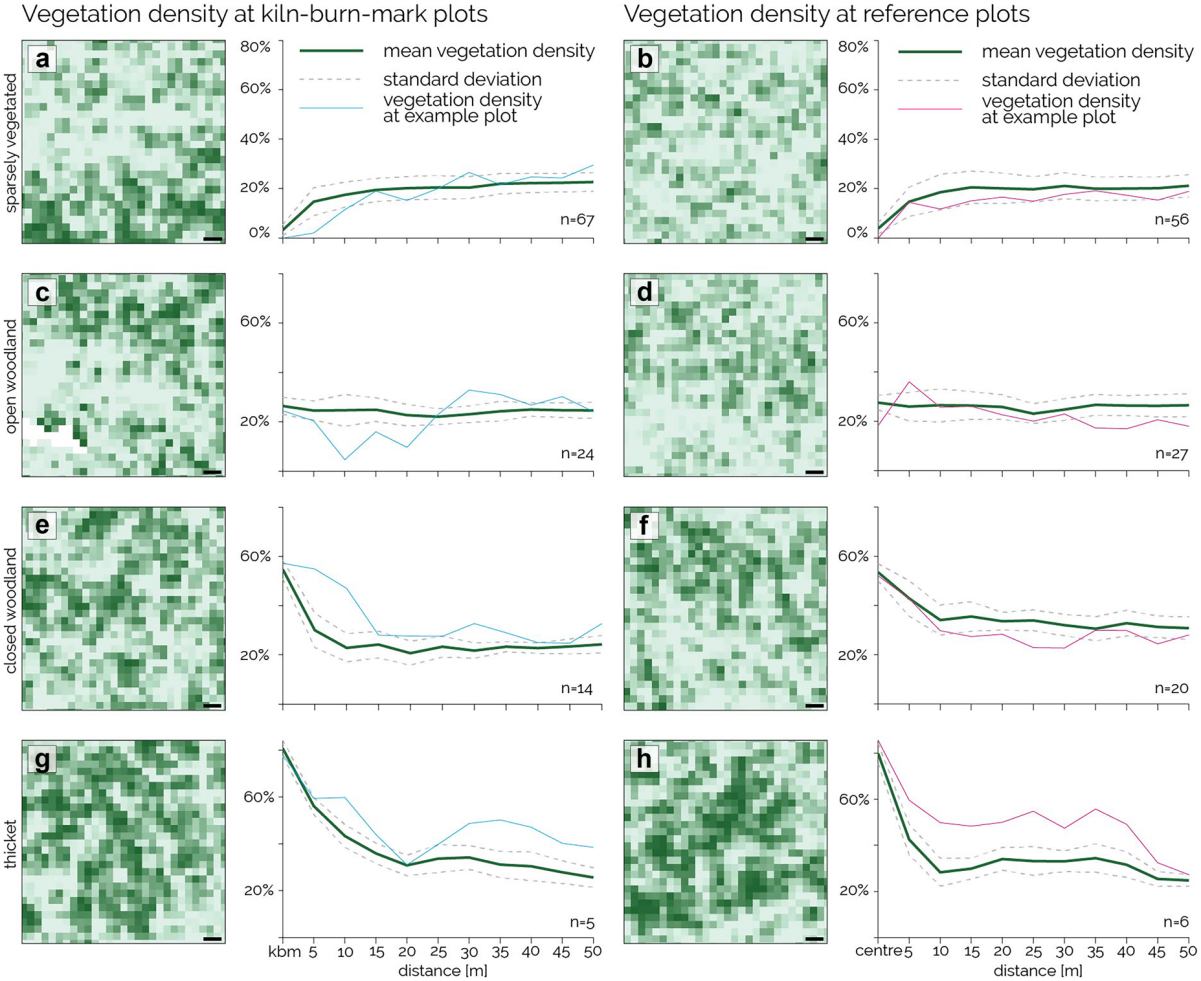
Mean vegetation density in relation to distance from plot centre. For KBM (**a**, **c**, **e**, **g**) and reference plots (**b**, **d**, **f**, **h**). The plots’ centres are located within the LULC class sparsely vegetated (**a**, **b**), open woodland (**c**, **d**), closed woodland (**e**, **f**), and thicket (**g**, **h**). The graphs show the mean vegetation density (solid green line), standard deviation (dotted grey line), and the values from the example plots (blue, pink lines) between the plots’ centre and up to 50-m distance in 5-m steps

The concentric ring analysis did not reveal significant patterns regarding vegetation density in relation to the increasing distance from kiln/KBM centres. Depending on the LULC class, mean vegetation density either increases (Fig. 4a) remains stable (Fig. 4c) or decreases (Fig. 4e, g) within the first 5 to 15 m around the centre.

After 25 m, it levels out at around 24% in all cases. Comparison with reference plots, where no kiln/KBM was detected in the centre, shows similar patterns. The vegetation density around sparsely vegetated plot centres rises from below 5 to 15% within 5 m surrounding the centre and then rises to 23% (KBM plots) and 21% (reference plots) between 45- and 50-m distance (Fig. 4a, b). In the class of open woodland, the mean vegetation density at the plot centre is slightly higher for reference plots (28%) than kiln/KBM plots (26%) and remains so in the surrounding. In the class of closed woodland, the vegetation density appears to decrease closer to the plot centre of kilns/KBMs compared to reference plots (Fig. 4e, f). While values at the centre are similar (55% for KBM and 53% for reference), they drop to 30% within the first 5 m surrounding a kiln/KBM but only to 43% surrounding a reference plot. Even between 45- and 50-m distance from the plot centre, average vegetation density is 7% surrounding a previously mapped kiln/KBM compared to reference plots (24% for KBM and 31% for reference). Within the LULC class of thickets, the effect is reversed as vegetation density around KBMs appears to decrease less rapidly than around reference plots (Fig. 4g, h). In both sets, values at the centre start high (81% at KBM, 80% at reference). However, while vegetation density decreases to 56% at the 5 m surrounding kiln/KBM plots, it drops to 42% around reference plots. After 15-m distance to the plot centre, the differences become less pronounced.

To validate the findings, the concentric ring analysis was repeated using SAVI values based on the WorldView-2 scene from 30 January 2019 and results show a similar pattern (SI 2).

### Other factors influencing vegetation structures

**Fig. 5:**
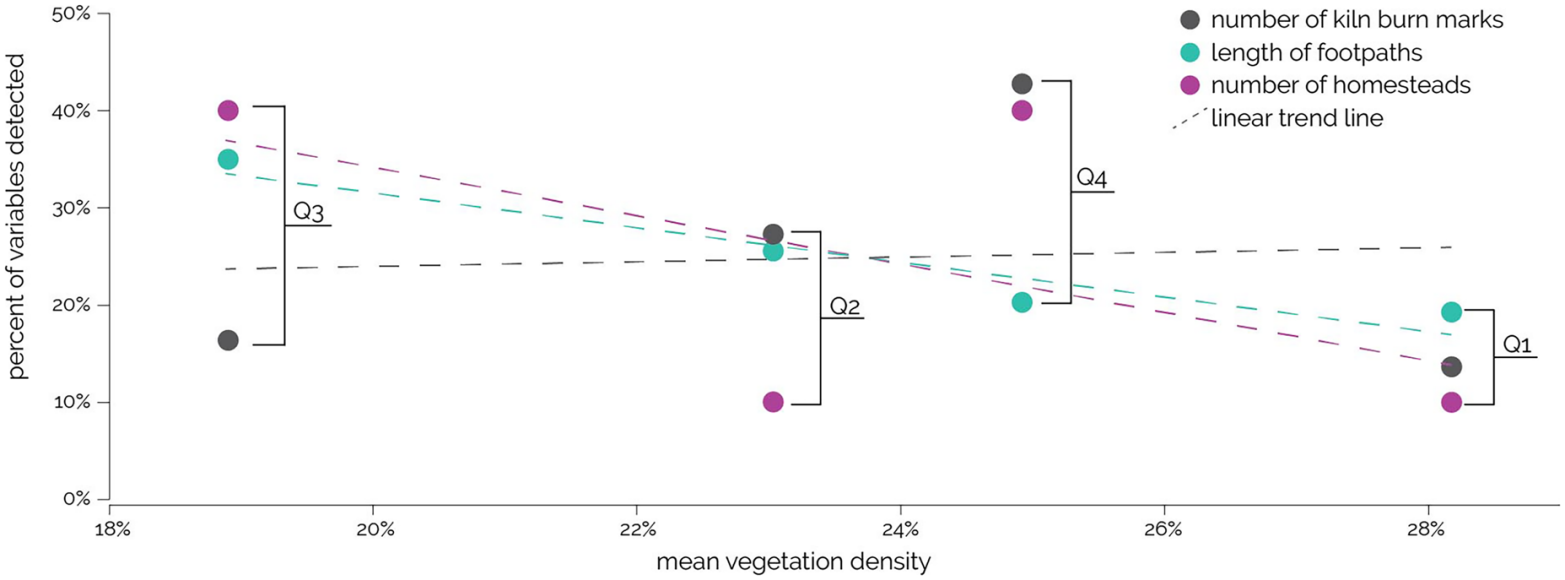
Other factors impacting vegetation characteristics. Percentage of mapped Kiln burn marks, footpaths, and homesteads (*y*-axis) in each of the four quadrants of the focus area in relation to the mean vegetation density (*x*-axis). While most kiln burn marks are located in Q4, it has the second highest mean vegetation density. Q1 with the fewest KBMs has the highest mean vegetation density but also the fewest homesteads and footpaths. Most homesteads and footpaths are located in Q3. This quadrant also has the lowest mean vegetation density and also the second lowest number of KBMs. Negative linear trends (dashed lines) exist between mean vegetation density and number of homesteads as well as footpaths. There is a weak positive correlation between mean vegetation density and number of kiln burn marks

To evaluate whether the general vegetation density can predict the number of settlements or KBMs, the focus area was split into four equal quadrants and the percentages of kilns/KBMs, homesteads, and (detectable) footpaths were measured. While a negative linear trend exists between mean vegetation density and number of homesteads as well as footpaths, there is a weak positive correlation between mean vegetation density and number of kilns/KBMs (Fig. 5). In the quadrant with the highest mean vegetation density (Q1, 28%), the number of homesteads (1) and length of footpaths (2.4 km) were low, whereas the quadrant with the lowest mean vegetation density (Q3, 19%) included the longest total length of footpaths (4.39 km) and the number of homesteads and structures were higher (ten structures within four homesteads, Fig. 5). However, the number of kilns/KBMs was the highest in quadrants with a medium vegetation density (Q2 and Q4). This indicates that vegetation density at the focus area is rather affected by other human activities than charcoal production.

## Discussion

The results of the concentric ring analysis revealed only small differences in vegetation characteristics between charcoal production sites (kiln/KBM plots) and reference plots. These minor effects were usually evened out after 20- to 25-m distance to the plot centre. The most noticeable trends detected in the UAS analyses are slightly lower vegetation density values (compared to reference plots) for KBM plots centred around dense woodlands in contrast to slightly higher vegetation density surrounding plots located in thickets. Of note, it is not necessarily expected to see an impact on vegetation density within the immediate surroundings in thickets, because it might be characterized by a lower availability of trees suitable for charcoal at these spots and thus, a larger radius might be needed to harvest wood. Yet, due to the very small subsample of plots within this LULC class, these results should be interpreted with caution.

A Landsat-based long-term change detection in the same study area could also not find a correlation between charcoal production and deforestation or forest degradation, which was rather linked to an increase in settlements and agricultural expansion (Petersen et al., 2021). These findings are in contrast to a study by Kouami et al. (2009), who found significantly lower woody plant density and diversity at plots exploited for charcoal production compared to those that were not. However, they acknowledge that their study did not take other anthropo-zoogenic activities into account, making it difficult to ascribe differences solely to charcoal production. The present study faces similar problems as it is difficult to precisely assign environmental effects to one specific activity. For example, it is not clear if the slightly lower vegetation density values around kiln/KBM plots in dense woodlands are a direct result from charcoal production or if the lower density in the surrounding was a reason to choose the spot as a production site due to easier accessibility in the first place. The second hypothesis is supported by unpublished reports produced by local informants. They have stated that the accessibility of an area plays an important role in choosing a production site. Areas of dense woodland, on the other hand, are generally avoided due to the presence of dangerous wild animals (Petersen et al., 2021).

Studies that found connections between deforestation and charcoal production further mention that in many cases, it is a side product of agricultural expansion (Doggart et al., 2020; Iiyama et al., 2015, 2017). This is supported by field observations conducted for this research project, which locate most larger kilns on cropland. In some study areas, however, the role of charcoal production appears to be less ambiguous. In Kitui County, Kenya, woodland degradation and selective logging were clearly linked to large-scale charcoal production but were unable to clearly identify effects of small-scale charcoal production (Kiruki et al., 2017). Similarly, Sedano et al. (2020) were able to reconstruct large-scale deforestation due to industrialized charcoal production but recognize that household-level production may go undetected with their methodology.

A major limitation of the UAS approach is the difficult separation of driving factors of environmental change. It cannot be determined unambiguously whether differences in vegetation density stem from charcoal production or other land-use practices. This common problem could have been mitigated by including additional focus areas or repeated flights (Aabeyir et al., 2016; Kouami et al., 2009). A reference plot without detectable human activity would be a useful addition. However, land tenure in the area and the Pokot principle which allows animals to be grazed (or browsed) on all Pokot land could complicate such study design and would require fencing and regular site monitoring (Bollig & Österle, 2008). Furthermore, the accuracy of the derived point cloud and subsequently the elevation and density models could be improved by a differential and/or a real time kinematics GPS, as well as cross-flight patterns (Gerke & Przybilla, 2016; Rizos et al., 2012).

Results from the SAVI analysis mostly correspond to those of the vegetation density. Smaller differences are because some trees had already defoliated due to water scarcity in January, which resulted in lower overall SAVI values. Though image matching and vegetation density is also affected by shed leaves, also, defoliated trees are recognized as vegetation and are thus reflected in the vegetation density model. On the other hand, the point cloud classification does not distinguish between built-up structures and vegetation for above ground points. Since only 25 small human-made structures are located in the focus area, they have been ignored. For other studies, it is advisable to manually classify the point cloud or add spectral information to exclude areas free of vegetation from the analysis.

During the project, several approaches have been considered to gain a detailed assessment of environmental effects of charcoal production on the vegetation structure. One included the automated delineation of kilns/KBMs based on remotely sensed imagery as several studies have already successfully employed a visual or automated detection of kilns and KBMs via VHR data (Bolognesi et al., 2015; Dons et al., 2015; Rembold et al., 2013; Sedano et al., 2016). At the site of the present study, this procedure proved not suitable. Average kiln size and output in the abovementioned studies are generally larger compared to those in Pokot Central (SI 3). The smaller size is most likely the most important factor, why KBMs and kilns are generally not visible in VHR imagery and can be rarely identified even in UAS imagery. However, also the common local practice to construct kilns beneath a large tree makes detection difficult. Furthermore, charcoal production estimates based on (automatically) detected kilns/KBMs, as done by Bolognesi et al. (2015), Rembold et al. (2013), and Dons et al. (2015) would most likely underestimate the real amount of charcoal produced in Pokot Central to a large extent. Dons et al. (2015) observed that 14 of 24 kilns had been re-erected after 6 months mostly to use unburnt leftovers from the initial kiln and that KBMs were usually not covered by vegetation for several years. Producers in the present study area, however, often reuse their production sites due to favourable conditions, and many KBMs are grown over by an herbal layer, as soon as the rainy season starts. Adding a thermal band to the investigation is likely to improve detection of active kilns but would not offer insights on KBMs. Thus, detection of kilns and KBMs based on satellite imagery and even based on UAS flights is only applicable for large-scale production areas. To detect small-scale production field information remains a research challenge.

The study design could further be improved using a UAS equipped with a multispectral camera and repeat flights over areas with various degrees of charcoal production and different agro-pastoral activities. Such an approach might greatly improve the vegetation analysis and enable an assessment of species composition and estimation of seasonal effects (Franklin & Ahmed, 2018; Kolarik et al., 2019; Prošek & Šímová, 2019). However, Kenyan UAS regulations were currently under revision during the study period and new ones were passed only in 2019 after field data collection had ended (KCAA, 2020; Koeva et al., 2020).

## Conclusion

The presented approach indicates no significant differences in vegetation density and SAVI between (former) production sites and reference plots. It furthermore shows that detected deviations are not easily attributed to one specific land-use practice. This finding is only valid for small-scale charcoal production sites but together with evidence from other studies, it calls for a rephrasing of the common narrative which depicts charcoal producers as solely responsible for forest cover loss. It shows that more research and new approaches are needed to evaluate the role of small-scale charcoal production in deforestation and forest degradation processes. Nevertheless, the potential of UAS in monitoring charcoal production is high if repeat flights and more diverse sampling areas are incorporated. Though UASs are a promising technology, inconsistent regulations and requirements are likely to prevent the full use of their potential in environmental applications. Clear regulations regarding conditions and costs for permits are needed to enable the development and implementation of new applications (Koeva et al., 2020; Stöcker et al., 2019).

## Supplementary Information

Below is the link to the electronic supplementary material.

Supplementary file1 (PDF 1709 KB)

## Data Availability

The datasets generated and analysed during the current study are not publicly available to protect the privacy of the local community and charcoal producers.

## Footnotes

1 Though “wood fuel” usually includes charcoal and firewood, a clear distinction between the two energy sources is essential due to the role of urbanization and commercialisation of charcoal (Mwampamba et al., 2013).

2 The exact location of the spot it not given to protect the anonymity of charcoal producers.

3 One bag of charcoal weighs approximately 50 kg (based on field measurements).
