# Supplementary material for: Using an unmanned aerial system to analyse environmental impacts of charcoal production on tropical savanna ecosystems in northwestern Kenya
Source: Environ Monit Assess. 2022 Jul 29;194(9):620. doi: 10.1007/s10661-022-10241-2 (PMC9338136; doi:10.1007/s10661-022-10241-2)
Supplement: Supplementary file 1 — Supplementary file1 (PDF 1709 KB) [file 10661_2022_10241_MOESM1_ESM.pdf]

Electronic Supplementary Material

Using an Unmanned Aerial System to analyse environmental impacts of charcoal production on tropical savanna ecosystems in northwestern Kenya

Maike Petersen[a*], Marcus Nüsser[a,b]

[a] SAI, Department of Geography | Heidelberg University
ORCID: 0000-0003-0156-9541

[b] HCE, Heidelberg Center for the Environment | Heidelberg University

marcus.nuesser@uni-heidelberg.de
ORCID: 0000-0002-8626-8336

[*] Corresponding author: Maike Petersen

Email: maike.petersen@ph-heidelberg.de

SI 1 Methods

Field and UAS survey

The UAS was equipped with Lithium-Polymer batteries, which allowed an average flight duration of around 20 minutes for each of the three available batteries. Pictures were collected in natural colour (RGB) by a 12-megapixel camera (FC220) mounted to a gimbal for stabilisation during flights. The camera was triggered either manually via the remote control or automatically based on a predefined pattern. A remote control connected phone and aerial system and allowed manual control in case of complications. Pix4D Capture was installed on a smart phone. Pix4D Capture facilitated pre-planned flight missions and automated image collection with pre-set image overlap and flight speed. A built-in GPS/GLONASS receiver tagged the images with their position and was further used to follow the predefined flightpath. To ensure flights within line of sight, the pre-planned flight missions were readjusted on the ground.

Image EXIF meta data was stored directly into the image files. Average flight altitude was 53 m above ground level (m a.g.l.), which resulted in a ground resolution of 1.63 cm per pixel. Images were taken at nadir view every two to four seconds at a medium speed and with a high degree of overlap (approximately 70 % side and 80 % frontal). Since the available Garmin GPS device can have positioning errors up to 10 m (Beekhuizen *et al.*, 2013) and the resulting model was not intended to be used in relation to locations outside of the focus area, no ground control points (GCP) were taken in the field. To enable comparison with the very high resolution (VHR) imagery, ten GCP were later added based on the 2019 WorldView-2 scene.

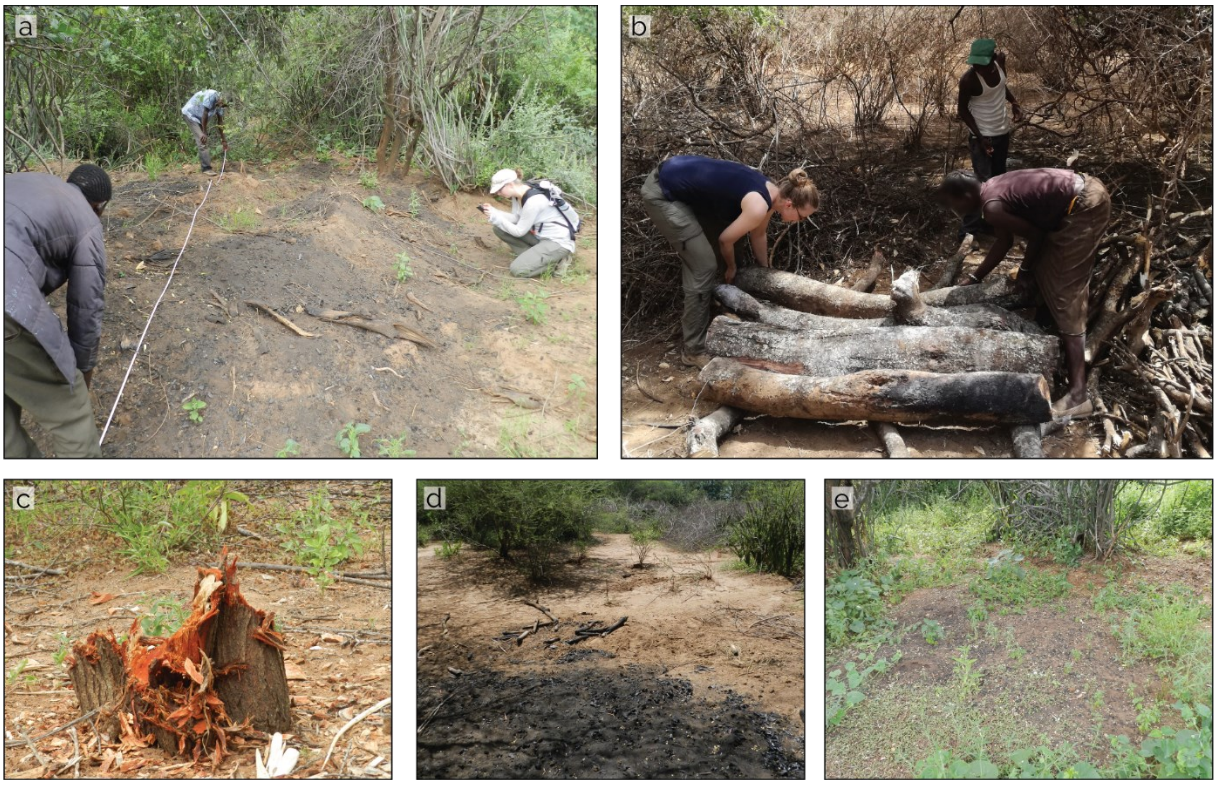

Fig. SI 1 Impressions from field measurements. **a.** Measuring and documenting a kiln burn mark in the focus area (C. Bergmann 2017). **b.** Construction of a kiln during charcoal measurements (C. Kortom 2017). **c.** A tree stump left behind after wood harvesting for charcoal production at one of the surveyed charcoal production sites. **d.** The burn mark from a recently harvested kiln. Most of the ash residues and some larger pieces of charcoal and burnt branches are still present. The dark colour clearly stands out from the light sandy soil. **e.** The burn mark of an older kiln. The ash residues have been washed out by rain and wind and vegetation is growing on the former production site.

36 ***Modelling vegetation density***

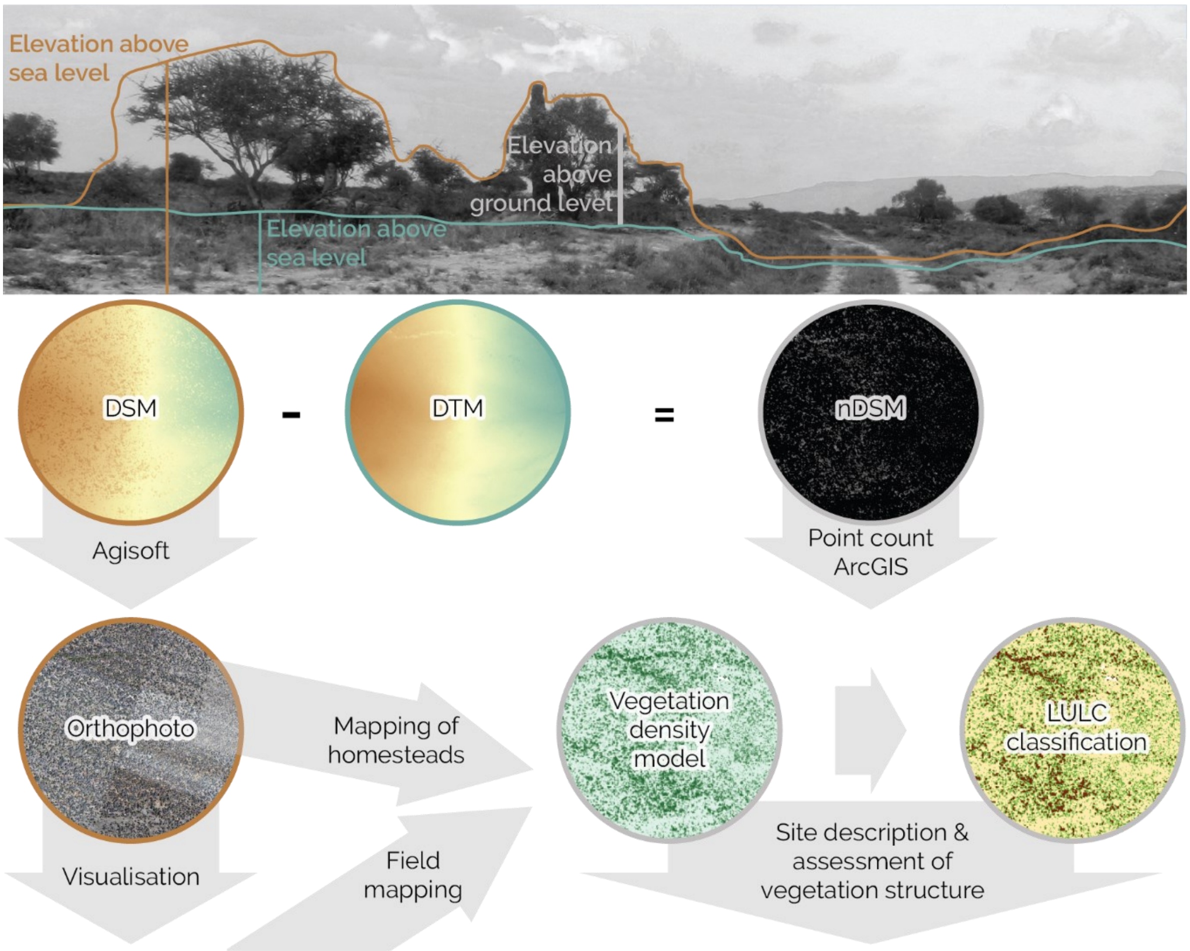

Fig. SI 2 Workflow of UAS data. The point clouds were classified into ground and non-ground points and processed into digital elevation models (DSM, DTM, and nDSM), which in turn were used to generate an orthophoto, a vegetation density model and a LULC classification. These outputs, in combination with field measurements were used for further analysis and visualisation.

37

SI 2 Results

Resulting models

Image matching of more than 4,000 images from several flights and further processing of the dense point cloud (Fig. SI 4a) resulted in a digital elevation model (DSM, Fig. SI 4b) with elevations between 925 m above sea level (m a.s.l.) and 970 m a.s.l., a pixel size of 10 cm and a point density of 100 points per m^2. Classification of the point cloud into ground points allowed generating a digital terrain model (DTM, between 920 m a.s.l and 970 m a.s.l.; Fig. SI 4c) and a subsequent subtraction of the DTM from the DSM produced an nDSM (Fig. 24d) with heights between 0 m and 14 m a.g.l. Some outliers resulted in single pixels with values below 0 m which were reclassified to 0 m as they only amounted to 0,24 % of raster cells. Ground elevation is lowest in the northeastern corner of the scene and rises towards the southwestern corner. Several riverbeds are visible in the DTM, especially in the northern half. In the southeastern corner of the nDSM lower height values correspond with the alluvial fan covered in V. nubica, while higher vegetation is located along the seasonal streams indicated in the DTM. The eastern half of the focus area shows lower vegetation heights compared to the western half and correlates with vegetation density, which is also lowest in the eastern half except for the northeastern border.

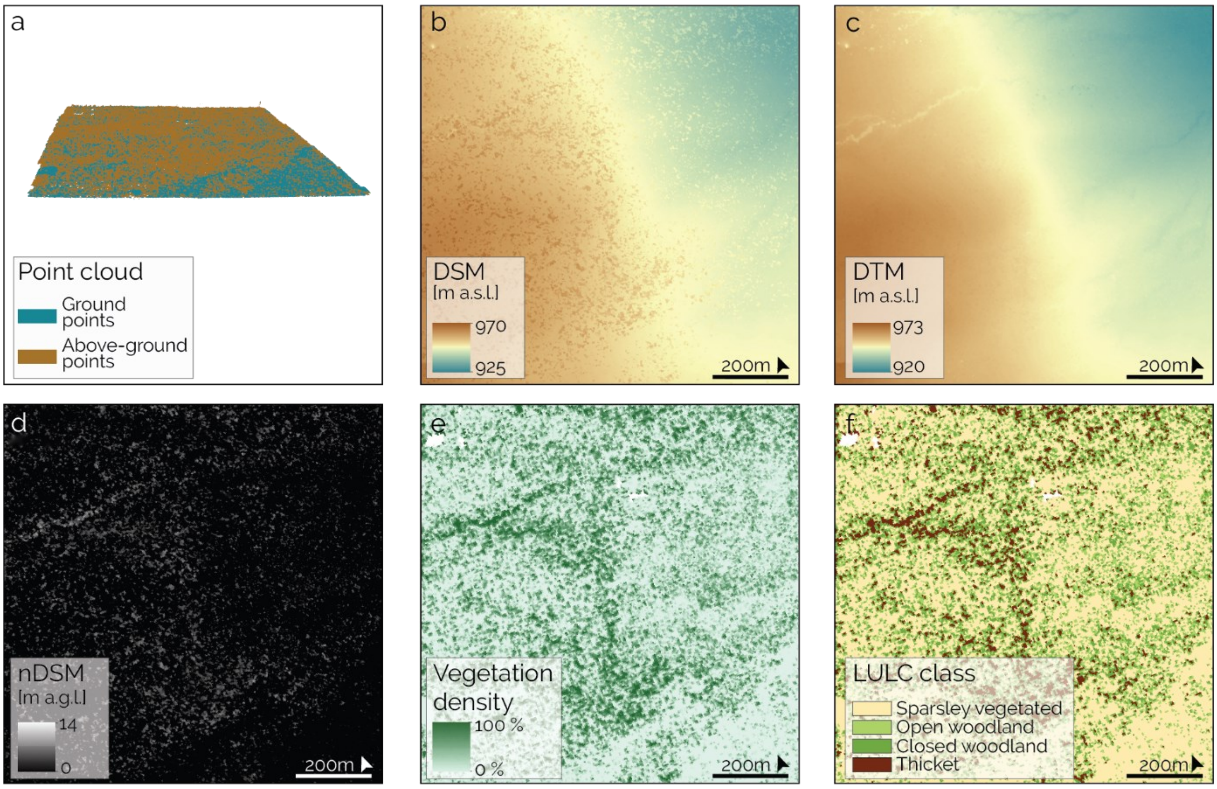

Fig. SI 3 Elevation models, vegetation density and LULC classes based on UAS images. Based on the classified point cloud (**a**) a DSM (**b**) and a DTM (**c**) were generated. Subtraction of the two resulted in an nDSM (**d**) which allowed calculation of the vegetation density (**e**) and classification into LULC classes (**f**). Ground elevation is lowest in the northeastern corner of the scene and rises towards the southwestern corner. In the DTM (**c**), empty riverbeds of seasonal streams along with higher vegetation density (**e**, **f**). are visible. In the southeastern corner of the nDSM lower height values correspond with the alluvial fan covered in *V. nubica* (**d**). The dominant LULC class based on vegetation density is *sparsely vegetated* (**f**).

Soil adjusted vegetation index in relation to distance from plot centre

In sparsely vegetated areas the SAVI increases between the plot centre and the first five meters around it and then remains at 0.18 (Fig. SI 4a&b). Within *open woodlands* it is constantly at a value around 0.18 (Fig. SI 4c&d), while for *closed woodlands* and *thicket* plots it drops from around 0.2 to 0.18 within the first 10 m to 15 m around the centre (Fig. SI 4e-h). No significant differences can be found between KBM and reference plots that are located within *sparsely vegetated* areas, *open woodlands*, and *thickets*. Only within *closed woodlands* the SAVI at KBM plots decreases more strongly within the first five metres around the centre and remains 0.01 below SAVI values around reference plots for the whole 50 m (Fig. SI 4e&f).

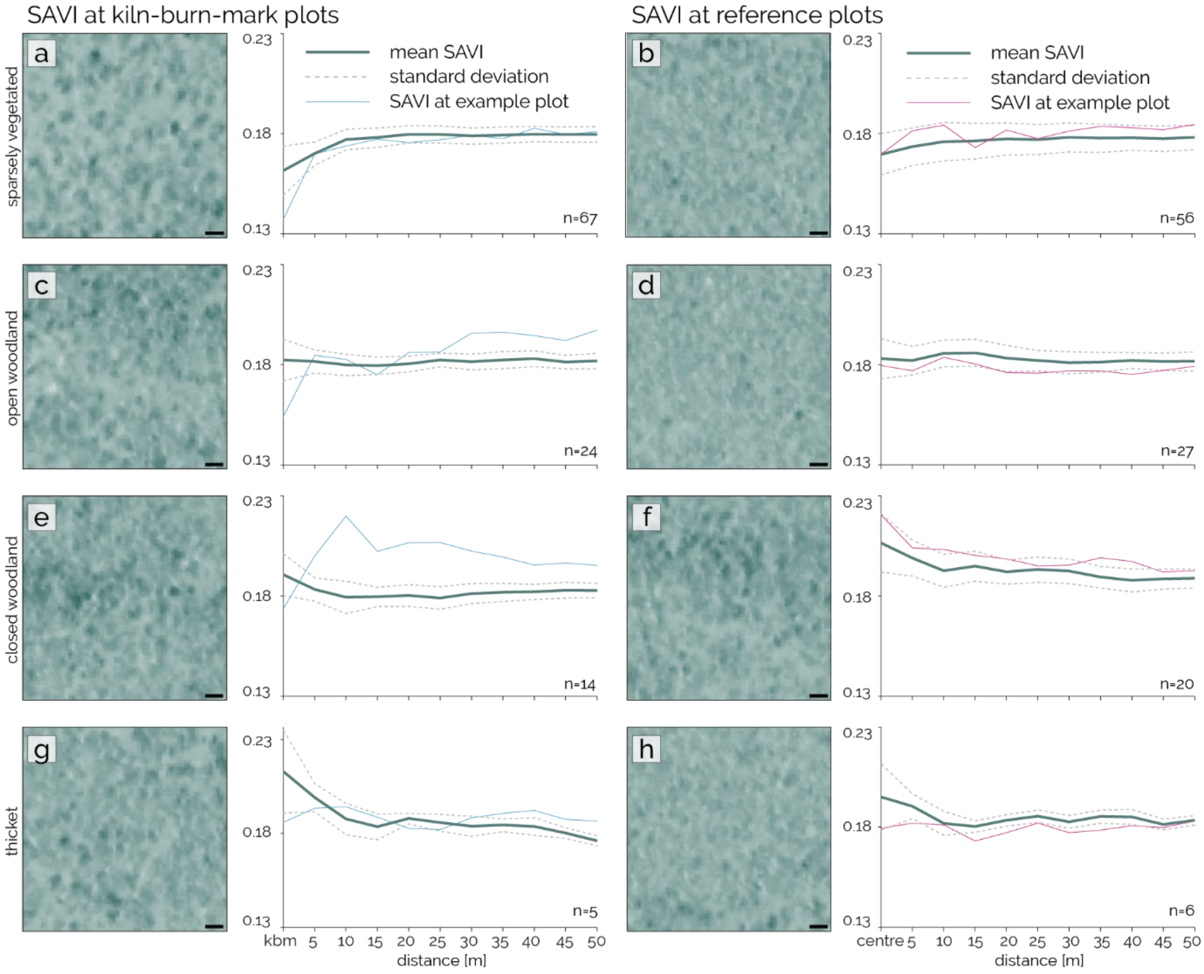

Fig. SI 4 Mean SAVI in relation to distance from plot centre. For KBM (**a, c, e, g**) and reference plots (**b, d, f, h**). The plots' centres are located within the LULC class sparsely vegetated (**a, b**), open woodland (**c, d**), closed woodland (**e, f**) and thicket (**g, h**). The graphs show the mean SAVI (solid green line), standard deviation (dotted grey line) and the values from the example plots (blue, pink lines) between the plots' centre and up to 50 m distance in 5 m steps.

SI 3 Discussion

Kiln sizes were reported at 31.7 m^2 and 5.7 m^2 (Dons *et al.*, 2015); 3 m – 9 m radius (Bolognesi *et al.*, 2015); 8.1 m length and 2.2 m width (Sedano *et al.*, 2016) and 12 m length (Rembold *et al.*, 2013) compared to average of 3.4 m^2 in Pokot Central. Kiln output was reported at 6.2 t and 0.7 t (Dons *et al.*, 2015); 0.7 t – 10.7 t (Bolognesi *et al.*, 2015); 1.6 t (Sedano *et al.*, 2016) and 1.1 t (Rembold *et al.*, 2013) compared to 0.2 t in Pokot Central.

SI References

Beekhuizen J, Kromhout H, Huss A, Vermeulen R. (2013) Performance of GPS-devices for environmental exposure assessment. Journal of Exposure Science & Environmental Epidemiology 23(5), 498–505. https://doi.org/10.1038/jes.2012.81

Bolognesi M, Vrieling A, Rembold F, Gadain H. (2015) Rapid mapping and impact estimation of illegal charcoal production in Southern Somalia based on WorldView-1 imagery. Energy for Sustainable Development 25, 40–49. https://doi.org/10.1016/j.esd.2014.12.008

Dons K, Smith-Hall C, Meilby H, Fensholt R. (2015) Operationalizing measurement of forest degradation: identification and quantification of charcoal production in tropical dry forests using very high resolution satellite imagery. International Journal of Applied Earth Observation and Geoinformation 39, 18–27. https://doi.org/10.1016/j.jag.2015.02.001

Rembold F, Oduori SM, Gadain H, Toselli P. (2013) Mapping charcoal driven forest degradation during the main period of Al Shabaab control in Southern Somalia. Energy for Sustainable Development 17(5), 510–514. https://doi.org/10.1016/j.esd.2013.07.001

Sedano F, Silva JA, Machoco R, Meque CH, Sitoe A, Ribeiro N, Anderson K, Ombe ZA, Baule SH, Tucker CJ. (2016). The impact of charcoal production on forest degradation: a case study in Tete, Mozambique. Environmental Research Letters 11(9), 094020. https://doi.org/10.1088/1748-9326/11/9/094020
